# Real-world characteristics and outcomes of ischemic stroke: a Peruvian multicenter analysis from the RES-Q registry

**DOI:** 10.3389/fstro.2026.1783830

**Published:** 2026-04-28

**Authors:** Marla Gallo-Guerrero, Wilfor Aguirre-Quispe, Camila Nicole Gallo-Lazarte, Sofía Stefanie Sanchez-Boluarte, Antero Peralta-Mestas, Liliana E. Rodriguez Kadota, Ivan José Aliaga Cordova, William Bayona Pancorbo, Manuel Moquillaza-Valle

**Affiliations:** 1 Neurology Department, Clinica Ricardo Palma, Lima, Peru; 2 Neurology Department, Hospital General Regional 180 IMMS, Tlajomulco de Zúñiga, Mexico; 3 Grupo de Investigación NEMECS: Neurociencias, Metabolismo, Efectividad Clínica y Sanitaria, Universidad Cientifica del Sur, Lima, Peru; 4 Universidad Peruana de Ciencias Aplicadas, Lima, Peru; 5 School of Medicine, Universidad Cesar Vallejo, Trujillo, Peru; 6 Neurology Department, Hospital Nivel IV Carlos Alberto Seguin Escobedo, Arequipa, Peru; 7 Neurology Department, Hospital Nacional Edgardo Rebagliati Martins, Lima, Peru; 8 Hospital Nacional Alberto Sabogal Sologuren, Lima, Peru; 9 Hospital Nacional Adolfo Guevara Velasco, Cusco, Peru

**Keywords:** ischemic stroke, Peru, registries, thrombolytic therapy, treatment outcome

## Abstract

**Objective:**

To characterize and compare clinical profiles, care times, and functional outcomes between thrombolyzed and non-thrombolyzed patients with acute ischemic stroke (IS) within the Peruvian healthcare system using real-world data from the RES-Q registry.

**Methods:**

A retrospective multicenter cohort study was conducted. Data from adult patients with acute IS registered between January 2023 and June 2025 across five hospital centers in Peru (Lima, Arequipa, Cusco) were analyzed. The primary exposure was intravenous thrombolysis (IVT). Primary outcomes were functional independence [modified Rankin Scale (mRS) 0–2] at discharge, symptomatic intracranial hemorrhage (sICH), and in-hospital mortality. Multivariable logistic regression was used to identify independent predictors of poor functional outcome and mortality.

**Results:**

Of 1,780 patients with IS, 252 (14.2%) received IVT. Despite presenting with more severe strokes (higher median NIHSS, *p* < 0.001), the IVT group had a significantly higher rate of functional independence at discharge (50.7% vs. 38.3%, *p* < 0.001). In multivariable analysis, IVT remained an independent predictor of good functional outcome (adjusted odds ratio for poor outcome: 1.30 for non-IVT group). The median door-to-needle time was 68 minutes. In-hospital mortality did not differ significantly between groups (7.14% vs. 5.76%, *p* = 0.389). Key independent predictors of poor functional outcome were higher NIHSS (aOR 1.20 per point, 95% CI 1.16–1.24, *p* < 0.001), age (aOR 1.01 per year, 95% CI 1.00–1.02, *p* = 0.017), and diabetes (aOR 1.45, 95% CI 1.05–1.99, *p* = 0.022). For mortality, significant predictors were NIHSS (aOR 1.25, 95% CI 1.19–1.32, *p* < 0.001) and diabetes (aOR 2.21, 95% CI 1.12–4.38, *p* = 0.022).

**Conclusion:**

This real-world multicenter study in Peru demonstrates that IVT is feasible and effective, associating with significantly better functional outcomes despite treatment of a more severely affected cohort. The study validates the safety profile of IVT in this setting and identifies critical areas for quality improvement, particularly in reducing door-to-needle times and addressing disparities in access to mechanical thrombectomy. Our findings support the expansion and optimization of acute stroke reperfusion protocols in Peru and similar resource-limited healthcare systems.

## Introduction

Ischemic stroke (IS) constitutes one of the leading causes of mortality and disability worldwide, representing a substantial burden for healthcare systems and economies (GBD 2021 Stroke Risk Factor Collaborators, 2024; GBD 2019 Stroke Collaborators, 2021; Strilciuc et al., 2021). In Latin America, the mean direct cost per patient with IS is estimated at 12,203 US dollars, varying significantly according to the treatment received and the country (Dittrich et al., 2025). This economic burden adds to the high disease burden, with an estimated loss of over 70 million disability-adjusted life years (DALYs) annually due to stroke-related death and disability (Feigin et al., 2025; GBD 2021 Stroke Risk Factor Collaborators, 2024), underscoring the critical need for effective and accessible interventions.

Intravenous thrombolysis (IVT) with alteplase is the cornerstone of acute IS treatment, with robust evidence demonstrating its efficacy in improving functional prognosis when administered within a window of up to 4.5 h from symptom onset (Hacke et al., 2008; Powers et al., 2019). Recent evidence has also demonstrated its efficacy in an extended time window under neuroimaging selection (Albers et al., 2018; Ma et al., 2019; Nogueira et al., 2018). However, this benefit must be carefully weighed against the risk of complications, the most feared being symptomatic intracranial hemorrhage (sICH) (Hacke et al., 2008).

Randomized clinical trials establish the efficacy of interventions under ideal conditions. However, it is through real-world observational studies (Real-World Evidence, RWE) that their effectiveness is confirmed in routine clinical practice (Qin et al., 2018). Global registries such as the World Stroke Registry (RES-Q) are crucial tools for this purpose, enabling the evaluation of guideline implementation, safety, and treatment outcomes across different contexts and healthcare systems (Fasugba et al., 2024).

Connecting the global problem with the local reality is essential. In Peru, IS represents a significant epidemiological burden. Population studies report an incidence of up to 7.05 per 1,000 inhabitants (Moyano et al., 2021), with in-hospital mortality that can reach approximately 11.5% (Labán-Seminario et al., 2022). Despite this high burden, the Peruvian healthcare system, fragmented into multiple subsystems [MINSA (Ministry of Health), EsSalud (Social Health Insurance), FFAA/PNP (Armed and Police Forces), and private], faces structural challenges for acute care. These include a marked concentration of neurologists and Stroke Units in the capital, Lima, as well as geographic and economic barriers that hinder timely access from other regions (Abanto et al., 2020).

Local evidence on the implementation of IVT is limited and reinforces this problem. A key single-center study conducted at the national neurological reference institute reported a thrombolysis rate of just 1.98% between 2009 and 2016, citing barriers in symptom recognition and in-hospital processes (Abanto et al., 2020). At the regional level, Ibero-American registries have confirmed that while IVT is safe in Latin America, its overall usage rates are low, often below 5%, and with prolonged door-to-needle times (Alonso de Leciñana et al., 2019). Therefore, a critical knowledge gap exists: there are no recent multicenter Peruvian studies that, using a standardized methodology like RES-Q, comprehensively compare the characteristics, management, and functional outcomes between thrombolyzed and non-thrombolyzed patients across a broader spectrum of hospitals.

Consequently, it is imperative to generate robust local evidence to guide health policies and optimize resources. The objective of this study is to use data from the RES-Q registry to characterize and compare the clinical-epidemiological profile, care times, and functional outcomes in a multicenter cohort of Peruvian patients with acute IS who received intravenous thrombolysis vs. those who did not receive this treatment.

## Methods

### Study design and population

A retrospective, analytical cohort study was conducted using data from the global stroke care quality registry RES-Q. All adult patients diagnosed with acute IS registered between January 2023 and June 2025 across five participating hospital centers in Peru were included. The study included hospitals from the capital, Lima, as well as from two other major cities in the country—specifically Arequipa and Cusco—allowing for the capture of the geographic and resource heterogeneity of the Peruvian healthcare system. The study was approved by the ethics committees of the participating centers.

### Data source and variables

The RES-Q database is used internationally in a standardized manner to collect information on stroke care. For this analysis, the following variables were extracted: Sociodemographic: Age and sex. Comorbidities: Hypertension, diabetes mellitus, atrial fibrillation, heart failure, coronary artery disease, hyperlipidemia, previous stroke (ischemic or hemorrhagic), and smoking. Stroke Characteristics: Severity at admission assessed by the Glasgow Coma Scale (GCS) and the National Institutes of Health Stroke Scale (NIHSS); pre-morbid functional status via the modified Rankin Scale (mRS); presence of wake-up stroke; and ASPECTS score on baseline neuroimaging. Stroke Etiology: Classified according to TOAST criteria (cardioembolic, large artery atherosclerosis, small vessel occlusion/lacunar, cryptogenic, other determined etiology). Treatment Received: Including administration of IVT and/or mechanical thrombectomy. Care Times: Onset-to-door time, door-to-imaging time, and door-to-needle time (for thrombolyzed patients).

### Definition of groups and outcome variables

Patients were divided into two cohorts based on whether they received intravenous thrombolysis (IVT Group vs. non-IVT Group). The primary exposure variable was IVT administration. The primary outcomes compared between groups were: Neurological Improvement: Defined by functional status at discharge via the mRS, with a good functional recovery defined as an mRS score of 0–2. Safety: Incidence of symptomatic intracranial hemorrhage (sICH). Functional Status at Discharge: mRS score. In-hospital Mortality.

### Statistical analysis

Quantitative variables are presented as means with standard deviation (SD) or medians with interquartile range (IQR) according to their distribution, assessed via normality tests (Shapiro-Wilk). Categorical variables are expressed as absolute frequencies and percentages. Baseline differences between the IVT and non-IVT groups were analyzed using Student's *t*-test or the Mann-Whitney U test for continuous variables, and the Chi-square (χ^2^) or Fisher's exact test for categorical variables.

To evaluate the independent association between thrombolysis and the outcomes of interest (neurological improvement, sICH, good functional recovery, and mortality), multivariable logistic regression models were performed, adjusting for potential confounding factors identified in the bivariate analysis or considered clinically relevant, such as age, initial stroke severity (NIHSS at admission), and significant comorbidities (e.g., hypertension, diabetes). Results are expressed as Odds Ratios (OR) with their 95% confidence intervals (95% CI). A *p-*value < 0.05 was considered statistically significant. All analyses were performed using Stata version 18.0 software (StataCorp LP, College Station, TX, USA).

## Results

A total of 2,703 patient records with a cerebrovascular diagnosis from five hospital centers were included. Of these, 1,780 (65.9%) were diagnosed with IS. The overall rate of intravenous thrombolysis use in the IS cohort was 14.2% (252 patients), with considerable variability between centers, ranging from 7.7% to 26.3%. The remaining 1,528 patients constituted the non-thrombolysis group for baseline and outcome comparisons (Table 1).

**Table 1:** Distribution of ischemic stroke by hospital center.

Hospital center	Records (*N*)	Ischemic stroke *n* (%)	Thrombolysis *n* (%)
Ricardo Palma Clinic	393	209 (53.2)	55 (26.3)
Adolfo Guevara Hospital	358	232 (64.8)	48 (20.7)
Carlos Alberto Seguin Hospital	195	182 (93.3)	35 (19.2)
Edgardo Rebagliati Hospital	857	611 (71.3)	72 (11.8)
Alberto Sabogal Hospital	900	546 (60.7)	42 (7.7)
**Total**	2,703	1,780 (65.9)	252 (14.2)

The comparison of baseline characteristics between patients who received thrombolysis (*n* = 252) and those who did not (*n* = 1,528) is presented in Table 2. No significant differences were observed between the groups regarding median age (74 vs. 73 years, *p* = 0.898) or sex distribution (58.7% vs. 58.3% male, *p* = 0.901).

**Table 2:** Characteristics of patients with ischemic stroke: thrombolysis vs. no thrombolysis.

Patient characteristics		Thrombolysis	No thrombolysis	*p*-value
		*n*: 252	*n*: 1,528	
Sociodemographic characteristics				
	Age, median (IQR)	74 (43–94)	73 (65–81)	0.898
	Age, mean ± SD	72.3 ± 13.4	72.5 ± 12.7	
	Sex, male, *n* (%)	148 (58.7)	891 (58.3)	0.901
Comorbidities, *n* (%)				
	Hypertension	146 (59.1)	991 (67.3)	**0.012**
	Diabetes mellitus	57 (23.1)	515 (34.9)	**< 0.001**
	Atrial fibrillation or flutter	28 (11.3)	180 (12.2)	0.693
	Previous stroke	43 (17.4)	251 (17.0)	0.877
	Previous ischemic stroke	43 (17.4)	243 (16.5)	0.722
	Coronary artery disease or myocardial infarction	11 (4.5)	47 (3.2)	0.309
	Smoking in the last 10 years	13 (5.3)	68 (4.6)	0.657
	Hyperlipidemia	9 (3.6)	82 (5.6)	0.211
	Congestive heart failure	5 (2.0)	40 (2.7)	0.529
	Venous thromboembolism	2 (0.9)	10 (0.9)	0.897
	Previous hemorrhage stroke	1 (0.4)	8 (0.5)	0.708
	Other comorbidities	18 (7.1)	88 (5.8)	0.389
Stroke characteristics				
	Wake up stroke	11 (4.4)	134 (8.8)	**0.018**
	Glasgow coma scale	14 (13–15)	15 (14–15)	**< 0.001**
	Prestroke mRS	0 (0–1)	0 (0–2)	**< 0.001**
	Discharge mRS	2 (1–3)	2 (1–4)	**0.012**
	NIHSS at admission	10 (7–16)	5 (3–10)	**< 0.001**
	Mechanical thrombectomy	9 (3.6)	11 (0.7)	**< 0.001**
	ASPECTS score	10 (8–10)	9 (8–10)	**< 0.001**
Etiology of ischemic stroke				
	Determined	238 (94.4)	1,323 (86.6)	**< 0.001**
	Large artery atherosclerosis	84 (35.3)	448 (32.3)	0.363
	Cardioembolism	83 (34.7)	329 (23.7)	**< 0.001**
	Cryptogenic	43 (18.1)	250 (18.0)	0.987
	Small vessel disease or lacunar infarction	27 (11.3)	286 (20.6)	**0.001**
	One of the more rare etiologies	3 (1.3)	30 (2.2)	0.362
	Onset to door	390 (344–447)	589 (419–804)	< 0.001
Time (minutes)	Door to imaging	32 (15–58)	66 (28–217)	< 0.001
	Door to needle	68 (40–111)	–	–
	Imaging to needle	37 (19–56.5)	–	–
Hospitalized in	Standard bed	135 (53.6)	996 (65.2)	Ref
	Monitored bed	46 (18.2)	300 (19.6)	0.500
	ICU/stroke unit	71 (28.2)	232 (15.2)	**< 0.001**
Main outcomes	mRS at discharge (0–2)	128 (50.7)	585 (38.3)	**< 0.001**
	NIHSS at discharge	4 (2–7)	4 (2–7)	0.601
	Mortality	18 (7.14)	88 (5.76)	0.389
	Hemorrhagic transformation	18 (7.14)	4 (0.26)	**< 0.001**

Bold values indicate statistical significance.

However, significant differences were identified in several comorbidities. Patients in the thrombolysis group had a significantly lower prevalence of hypertension (59.1% vs. 67.3%, *p* = 0.012) and diabetes mellitus (23.1% vs. 34.9%, *p* < 0.001) compared to the non-thrombolysis group. There were no significant differences in other comorbidities such as atrial fibrillation, heart failure, or coronary artery disease.

Regarding stroke characteristics, the thrombolysis group had a lower proportion of wake-up stroke (4.4% vs. 8.8%, *p* = 0.018). At admission, this group showed a lower median Glasgow Coma Scale score (14 vs. 15, *p* < 0.001) and a worse pre-morbid functional status according to the mRS (*p* < 0.001). The ASPECTS score on baseline computed tomography was significantly higher in the thrombolysis group (median 10 vs. 9, *p* < 0.001). As expected, the use of mechanical thrombectomy was significantly higher in the thrombolysis group (3.6% vs. 0.7%, *p* < 0.001).

Regarding stroke etiology, thrombolysis was associated with a higher probability of having a determined etiology (94.4% vs. 86.6%, *p* < 0.001), specifically a higher frequency of cardioembolic stroke (34.7% vs. 23.7%, *p* < 0.001), and a lower frequency of small vessel disease or lacunar infarction (11.3% vs. 20.6%, *p* = 0.001).

### Functional outcomes

The proportion of patients who achieved functional independence (mRS 0–2) at discharge was significantly higher in the thrombolysis group (50.7%) compared to the non-thrombolysis group (38.3%) (*p* < 0.001).

**Figure 1:**
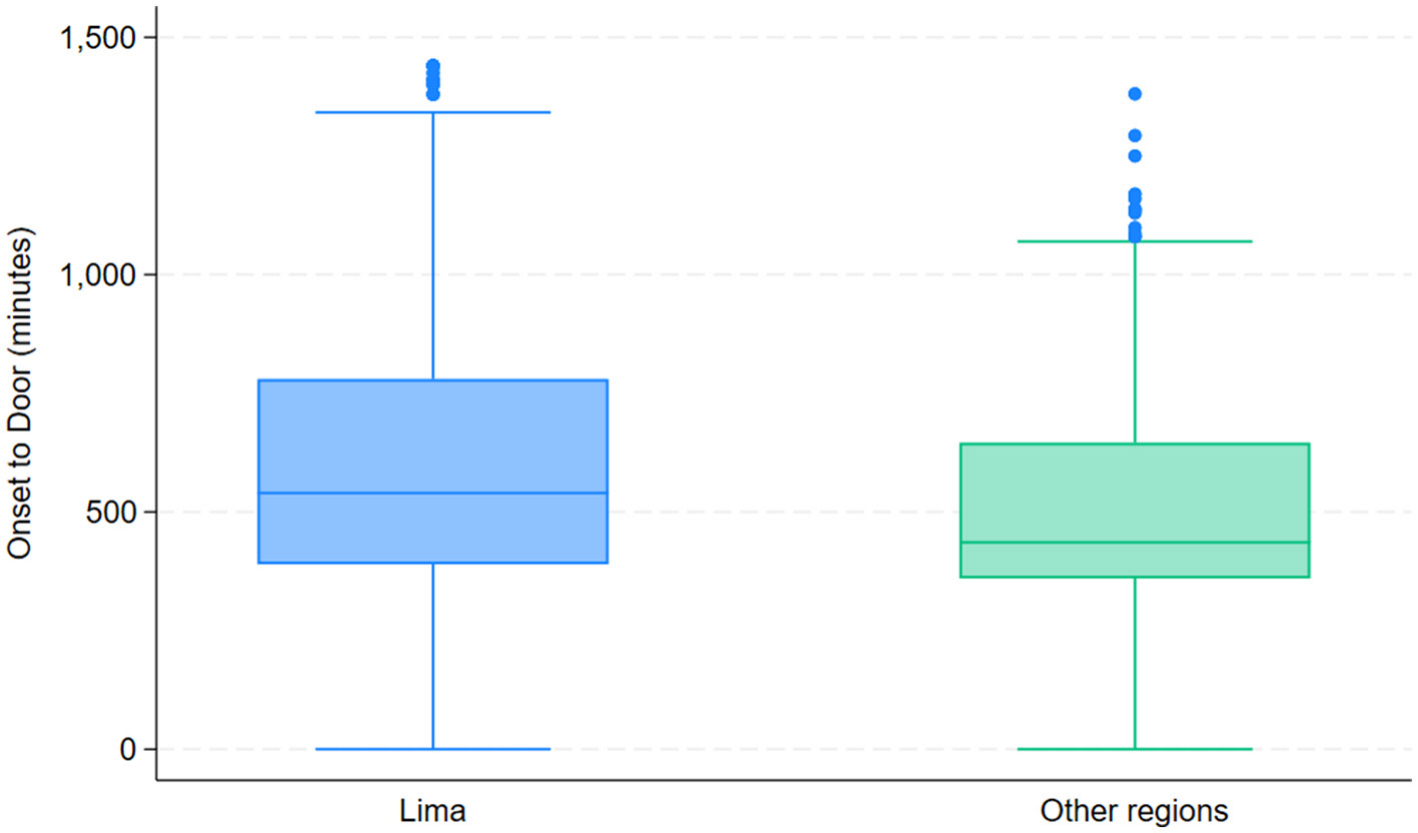
Time from Symptom Onset to Hospital Arrival (Onset-to-Door Time) by region.

**Figure 2:**
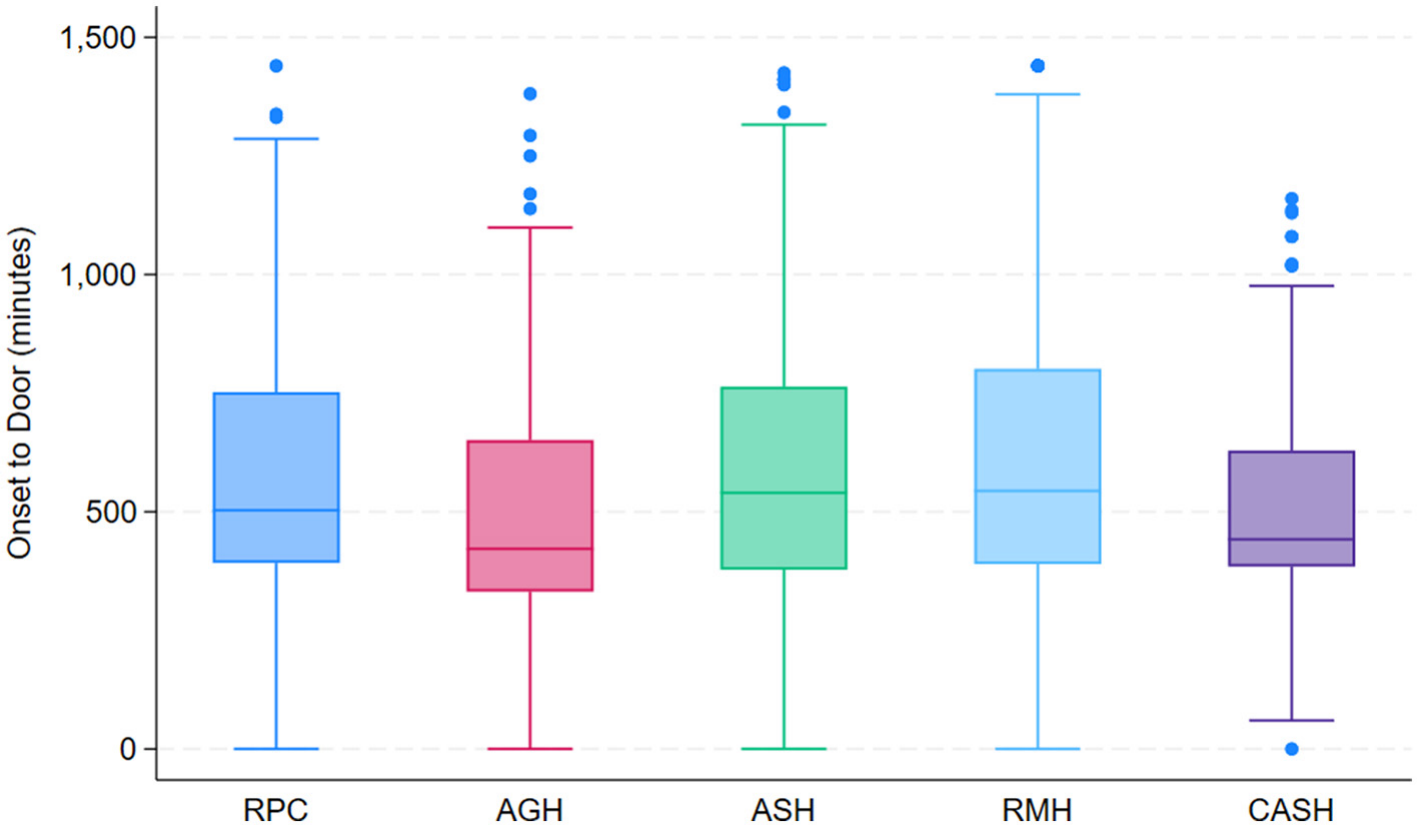
Time from Symptom Onset to Hospital Arrival (Onset-to-Door Time) by center.

The detailed distribution of mRS scores at discharge is presented in Figure 1. A visual comparison reveals distinct patterns between the two groups. The thrombolysis group exhibited a higher combined proportion of patients with excellent outcomes (mRS 0 and 1) and a lower proportion with moderate to severe disability (mRS 3–5). The overall difference in the distribution of mRS scores between the treatment groups was statistically significant (*p* < 0.001). Data on functional status at 3 months were available for 446 patients (74 thrombolyzed and 372 non-thrombolyzed). The distribution of mRS scores at this follow-up is shown in Figure 2. The thrombolysis group maintained a more favorable functional profile, with a higher combined proportion of patients achieving functional independence (mRS 0–2: 64.9% vs. 55.3% in the non-thrombolysis group). Notably, the thrombolysis group showed a higher proportion of patients with no symptoms (mRS 0: 20.3% vs. 14.2%) and a markedly lower proportion of patients with moderate-severe disability (mRS 3–5: 12.3% vs. 24.4%). The mortality rate (mRS 6) at 3 months was 23.0% in the thrombolysis group and 20.2% in the non-thrombolysis group.

The overall in-hospital mortality of the cohort was 5.9% (*n* = 106). When stratified by treatment group, mortality in the thrombolysis group was 7.14% (*n* = 18/252), while in the non-thrombolysis group it was 5.76% (*n* = 88/1,780) (*p* = 0.389).

### Care processes and quality metrics

**Figure 3:**
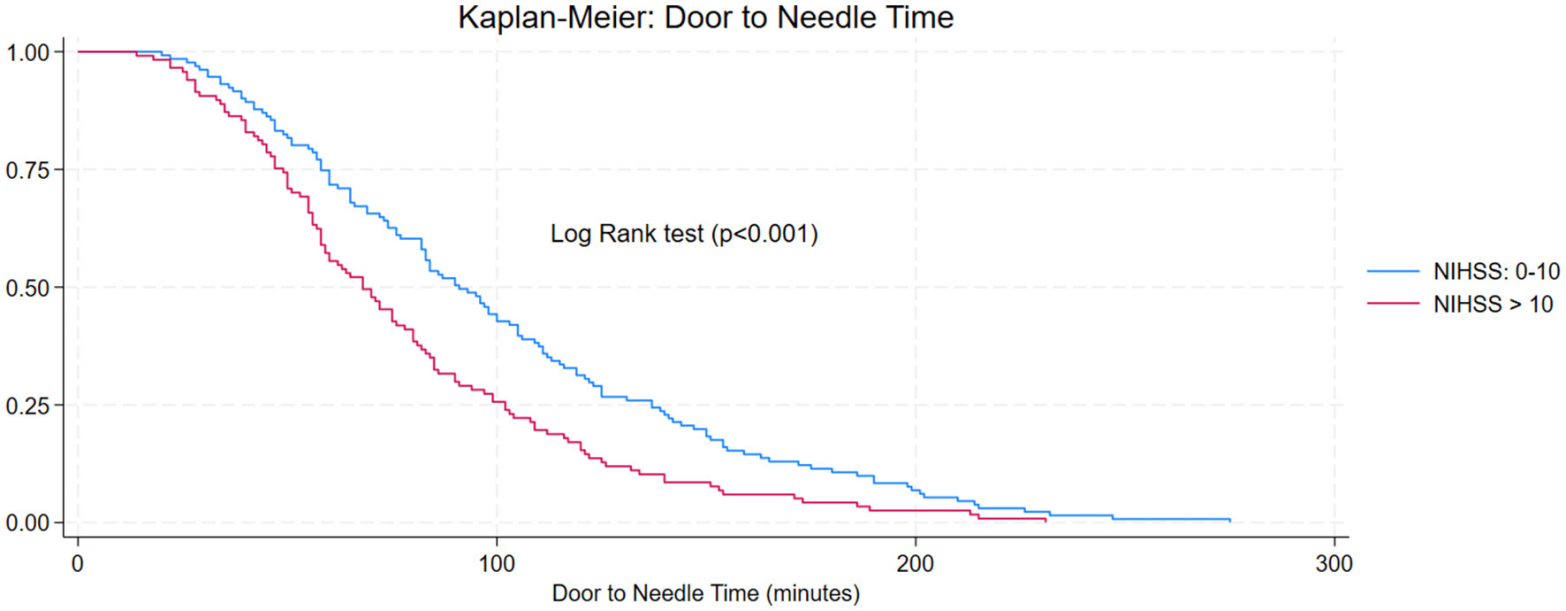
Door-to-Needle Time by Baseline Stroke Severity.

**Figure 4:**
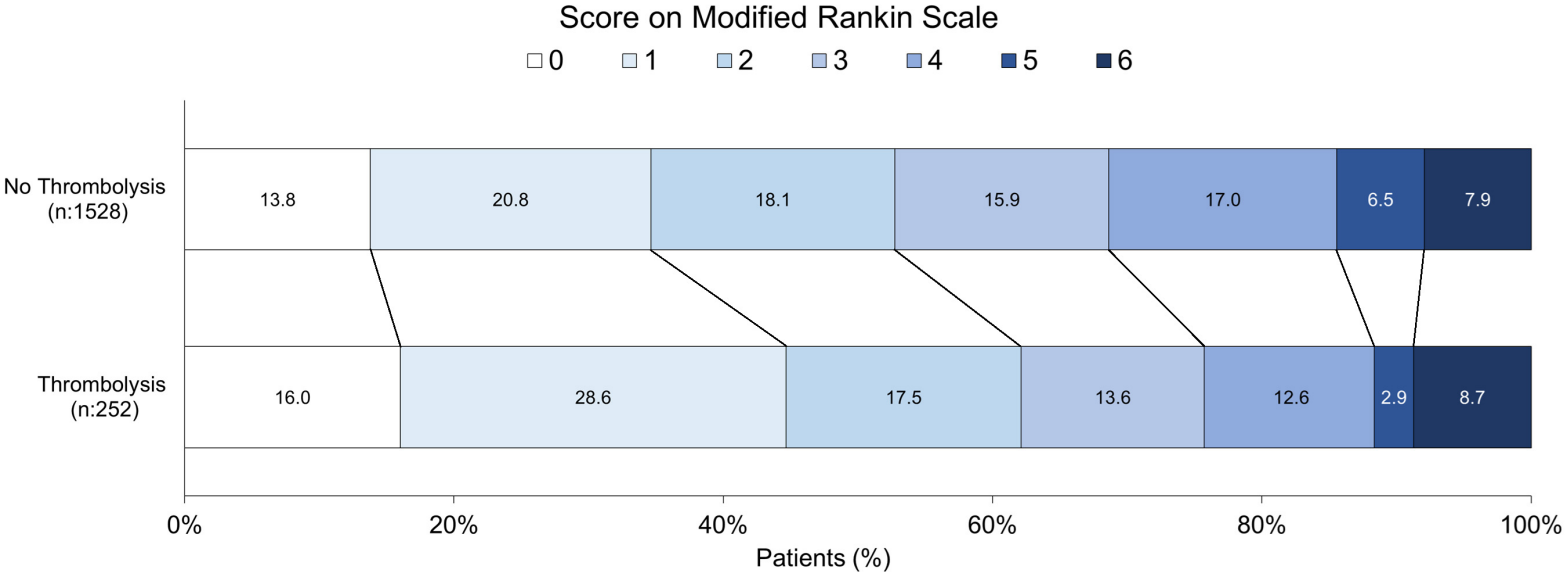
Distribution of modified Rankin Scale scores at hospital discharge by treatment group.

**Figure 5:**
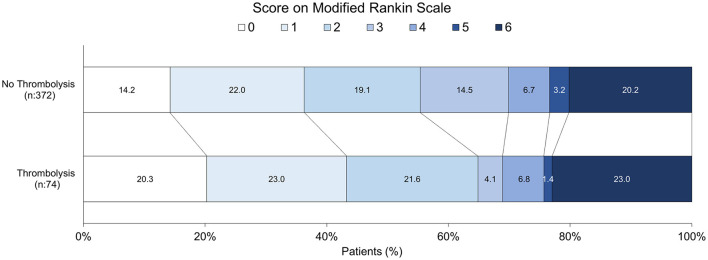
Distribution of modified Rankin Scale scores at 3 months by treatment group.

Analysis of care times revealed significant differences between Lima and the other included cities (Arequipa and Cusco). The median time from symptom onset to hospital arrival was 540 min (IQR: 390–780) in Lima vs. 436 min (IQR: 360–646) in the other cities (*p* < 0.001) (Figures 3, 4). Notable differences were also observed in in-hospital care times. The door-to-imaging time was significantly shorter in the thrombolysis group (median 32.5 min vs. 66 min, *p* < 0.001). The median door-to-needle time in thrombolyzed patients was 68 min (IQR 40–111), and the imaging-to-needle time was 37 min (IQR 19–56.5). The association between stroke severity at admission and the speed of care was assessed using Kaplan-Meier curves for door-to-needle time. A significant difference was observed between patients with NIHSS ≤ 10 and those with NIHSS > 10 (Log-rank test, *p* < 0.001). The median door-to-needle time was notably shorter in the NIHSS > 10 group, indicating that patients with more severe strokes were treated with higher priority (Figure 5).

Hospital destination following initial evaluation in the emergency department varied according to resource availability and clinical severity. Of the total patients, 17.1% (*n* = 303) were admitted to a Stroke Unit / ICU, 19.4% (*n* = 346) to monitored beds, and 63.5% (*n* = 1,131) to a standard hospital ward. Comparing by treatment group, patients who received thrombolysis had a higher probability of being admitted to a Stroke Unit / ICU (28.2% vs. 15.2%, *p* < 0.001) compared to those who did not receive thrombolytic therapy. This difference reflects both the need for close post-thrombolysis monitoring and the greater availability of specialized units in centers that administer reperfusion therapy.

### Subgroup analyses

In sub-analyses of patients on prior anticoagulant therapy (*n* = 152), warfarin was the most common agent (46.7%), followed by rivaroxaban (26.9%) and apixaban (19.7%) (Table 3). In this subpopulation, cardioembolic etiology was overwhelmingly predominant (76.4%), and most had atrial fibrillation (75.8%) (Table 4). The mean NIHSS score at admission in this group was 10.19 (±7.07).

**Table 3:** Ischemic stroke in anticoagulant users.

Type of anticoagulant	Frequency *N*: 152	Percentage (%)
Warfarin	71	46.7
Rivaroxaban	41	26.9
Apixaban	30	19.7
Dabigatran	5	3.2
Heparin	1	0.6
Other	4	2.6

**Table 4:** Etiology of ischemic stroke in anticoagulant users.

Characteristic	Means and proportions
Age, years (mean ± SD)	76.25 ± 12.14
Etiology of ischemic stroke, *n* (%)	
Cardioembolism	113 (76.40)
Atrial fibrillation	119 (75.80)
Cryptogenic	12 (8.10)
Atherosclerosis	8 (5.40)
Unknown etiology	7 (4.70)
Lacunar	5 (3.40)
Other etiology	4 (2.70)
NIHSS score (mean ± SD)	10.19 ± 7.07

Of all patients evaluated, 1.12% (*n* = 20) received mechanical thrombectomy as reperfusion therapy, either as standalone treatment or in combination with intravenous thrombolysis. It is important to note that, during the study period, only one center in the participating network had the capacity to perform mechanical thrombectomy: Ricardo Palma Clinic in Lima.

### Factors associated with functional outcome and mortality

In multivariable logistic regression analyses, we identified distinct predictors for poor functional outcome and in-hospital mortality (Table 5). For poor functional outcome (mRS 3–6 at discharge), each point increase in the NIHSS score was associated with a 20% higher odds (aOR 1.20, 95% CI 1.16–1.24, *p* < 0.001). Similarly, increasing age (aOR 1.01 per year, 95% CI 1.00–1.02, *p* = 0.017) and diabetes (aOR 1.45, 95% CI 1.05–1.99, *p* = 0.022) were significant independent predictors of poor functional outcome. Hypertension showed a non-significant trend toward association with worse outcomes (aOR 1.34, 95% CI 0.96–1.85, *p* = 0.080). It is noteworthy that onset-to-door time showed no significant association with functional outcomes.

**Table 5:** Multivariable logistic regression analysis of factors associated with functional outcome and mortality.

Patient characteristics	Poor functional outcome at discharge (mRS 3–6)		Death	
	**aOR (95% CI)**	***p*** **-value**	**aOR (95% CI)**	***p*** **-value**
Onset to door (per minute)	0.99 (0.99–1.00)	0.581	0.99 (0.99–1.00)	0.467
NIHSS (per point)	1.20 (1.16–1.24)	**< 0.001**	1.25 (1.19–1.32)	**< 0.001**
Age (per year)	1.01 (1.00–1.02)	**0.017**	1.00 (0.98–1.03)	0.499
Diabetes	1.45 (1.05–1.99)	**0.022**	2.21 (1.12–4.38)	**0.022**
Hypertension	1.34 (0.96–1.85)	0.080	1.00 (0.48–2.08)	0.996

mRS, modified Rankin Scale; aOR, adjusted odds ratio; CI, confidence interval; NIHSS, National Institutes of Health Stroke Scale.

Poor functional outcome was defined as mRS score 3–6 at hospital discharge. Bold values indicate statistical significance.

For in-hospital mortality, the NIHSS score again emerged as the strongest predictor, with each point increase associated with a 25% higher odds of death (aOR 1.25, 95% CI 1.19–1.32, *p* < 0.001). Diabetes was also significantly associated with mortality (aOR 2.21, 95% CI 1.12–4.38, *p* = 0.022), representing more than a two-fold increased risk. Neither age, hypertension, nor onset-to-door time showed significant associations with mortality in the adjusted model.

## Discussion

Our study represents one of the first and most extensive multicenter analyses conducted in Peru using standardized data from the RES-Q registry to compare baseline characteristics and functional outcomes of patients with acute IS who received IVT vs. those who did not. Our findings demonstrate that the implementation of IVT in the resource-limited setting of Peru is feasible and, crucially, is associated with a significantly higher proportion of patients achieving functional independence (mRS 0–2) at discharge. This benefit is observed despite the fact that the thrombolyzed patient group initially presented with more severe IS, validating the treatment's effectiveness in our context. Nevertheless, this analysis also identifies critical areas for improvement in the quality of care, notably in reducing the door-to-needle time (DNT).

The significant differences observed in baseline characteristics between thrombolyzed and non-thrombolyzed groups are highly informative and reflect an appropriate clinical selection process in the real-world practice setting. It is expected that the IVT group had a higher mean National Institutes of Health Stroke Scale (NIHSS) score and a lower Glasgow Coma Scale (GCS) score at admission. This pattern is consistent with international literature, where IVT is prioritized in more severe IS due to the greater absolute benefit of treatment in terms of reperfusion and recovery in this subgroup (Hacke et al., 2008; Powers et al., 2019).

The lower prevalence of hypertension and diabetes mellitus in the IVT group could be interpreted in several ways. On one hand, it might reflect stricter selection, excluding patients with uncontrolled vascular risk factors that could increase the risk of hemorrhage. On the other hand, the higher frequency of cardioembolic etiology and lower frequency of small vessel disease in the IVT group align with clinical practice guidelines (Kernan et al., 2014).

The main efficacy finding is robust: the proportion of patients who achieved a favorable functional outcome (mRS 0–2) at discharge was significantly higher in the IVT group (50.7% vs. 38.3%). This result demonstrates that, despite the greater initial severity of IS in the treatment group, IVT increased the odds of achieving functional independence at discharge by nearly 30% compared to the non-thrombolyzed population. This favorable response rate aligns with the results of large clinical trials and global real-world registries (Qin et al., 2018; Betts et al., 2017), thus confirming the successful transfer of efficacy demonstrated in trials to Peruvian clinical practice.

Analysis of care times reveals both strengths and weaknesses in Peruvian centers. The median door-to-imaging time was very good (32.5 min), indicating the existence of efficient protocols for triage, activation of the Code Stroke, and acquisition of neuroimaging. This achievement is crucial, as rapid triage is the first step in minimizing delays (Ghafoor et al., 2018).

However, the median DNT of 68 min exceeds the 60-min target established by international guidelines (Hacke et al., 2008). This difference suggests that the primary delay occurs after neuroimaging is obtained. The potential causes for this delay may be multifactorial, including medication preparation, waiting for family or financial authorization—a common problem in resource-limited health systems (Abanto et al., 2020)—or the need for a more in-depth neurological evaluation to confirm eligibility. Therefore, reducing the imaging-to-needle time is the most concrete and critical opportunity for improvement in stroke quality initiatives in Peru.

In terms of safety, no significant difference was found in in-hospital mortality between the groups, which is consistent with literature indicating that the main benefit of thrombolysis is the reduction of disability rather than short-term mortality.

The mortality rate observed in our study (5.9%) falls within the range reported in local literature. Previous studies in Peru have reported in-hospital mortality rates of up to 11.5% in national trend analyses (Labán-Seminario et al., 2022), figures comparable to our findings. The mortality observed in our thrombolysis group (7.14%) is consistent with international registries reporting rates between 10 and 11.4% in thrombolyzed patients (Heuschmann et al., 2004; Bateman et al., 2006), and suggests that thrombolytic therapy, when administered appropriately, does not increase mortality and may be associated with better functional outcomes. This finding suggests that any increase in the risk of hemorrhage associated with IVT is offset by the benefit of functional recovery, a pattern consistently observed in meta-analyses of global registries (Qin et al., 2018).

Regarding the strong association between diabetes and both poor functional outcome and mortality, this highlights the critical importance of this modifiable risk factor. Diabetic patients had a 45% higher probability of poor functional recovery and more than double the probability of death, emphasizing the need for optimized glycemic control in stroke prevention and acute management protocols. This finding is particularly relevant for Peru, where the prevalence of diabetes continues to rise and represents a substantial public health burden.

Interestingly, the onset-to-door time showed no significant association with any of the outcomes in our adjusted models. This contrasts with the conventional emphasis on early presentation, but may reflect several contextual factors: many patients may have presented outside the therapeutic window, the overwhelming effect of initial stroke severity may eclipse the benefits of time, or system-level factors in Peruvian healthcare delivery might attenuate the time-outcome relationship. This finding warrants further investigation into specific barriers to timely reperfusion therapy in our setting.

Regarding hemorrhagic transformation after thrombolysis, this occurred in 7.14% of cases, compared to 0.26% in those who did not receive thrombolysis, a finding similar to the literature, which ranges between 5% and 7% (Palaiodimou et al., 2025). This reinforces the safety profile of IVT even in patients with higher baseline severity, such as those included in the treatment group.

The rate of IVT use reported in this study (14.2%) is notably higher than historical rates previously reported in Peru and in many Latin American countries, which are often below 8% (Alonso de Leciñana et al., 2019). We argue that this higher rate reflects the ability of centers that actively participate in a quality registry like RES-Q to optimize their care processes and adhere to standardized protocols. This model of continuous improvement through the monitoring and feedback provided by registries has proven to be scalable to other hospitals in the country. However, the variability between centers (with IVT rates ranging from 7.7% to 26.3%) underscores the need to address local factors, such as in-hospital logistics, leadership in the stroke unit, and resource availability, to achieve more homogeneous care at the national level.

Our findings also suggest several improvement strategies that could be implemented to further enhance acute stroke care in Peru. First, although door-to-needle times were shorter for more severe strokes, the overall median of 68 minutes remains above the 60-minute benchmark. Systematic prehospital notification systems and in-hospital code stroke protocols with continuous quality monitoring have been shown to reduce treatment delays in Latin America; for instance, the SITS-SIECV Ibero-American registry demonstrated that centers with dedicated stroke teams and prehospital notification achieved significantly shorter door-to-needle times (Alonso de Leciñana et al., 2019). Second, the very low rate of mechanical thrombectomy (1.12%) and its concentration in a single center highlights a critical access gap. Regional hub-and-spoke networks, as implemented in Brazil, have proven effective in expanding access to endovascular treatment by coordinating rapid transfer from non-endovascular centers (Ouriques Martins et al., 2019). Third, the high proportion of patients admitted to standard wards (63.5%) underscores the need to expand dedicated stroke units, which have been shown to reduce death and dependency even in resource-limited settings (Langhorne and Ramachandra, 2020). Fourth, the strong association of diabetes with poor outcomes calls for integrating secondary prevention programs into acute care. Collectively, these strategies align with regional recommendations from the World Stroke Organization and the Latin American Stroke Organization for optimizing stroke systems in middle-income countries (Lindsay et al., 2014; Ouriques Martins et al., 2019).

Regarding mechanical thrombectomy, this limitation in availability reflects a critical gap in acute stroke care in Peru, particularly for patients with large vessel occlusions who could benefit from this intervention. The lack of availability of this technology limits equitable access, especially for patients in the public health system and those residing outside the capital.

## Limitations

Our study has limitations inherent to its observational design, which prevents establishing direct causality between IVT and improved functional prognosis, limiting us to reporting associations. The potential for residual confounding factors, despite adjustments, is a consideration. Furthermore, the included centers are likely referral hospitals with greater resources and established stroke protocols, so the results may not be fully generalizable to rural or lower-complexity hospitals in Peru. The absence of long-term follow-up data (mRS at 3 months) limits the ability to compare our results with the gold standard in the literature. Finally, the difficulty in discerning causality in findings such as the lower prevalence of HTN in the IVT group is a recognized limitation.

## Conclusion and implications

In conclusion, this multicenter Peruvian study strongly supports the implementation of intravenous thrombolysis as a highly effective and safe treatment for IS in Peruvian hospitals. Our results validate that IVT is associated with a better functional prognosis at discharge, even in patients with severe IS. The implications for clinical practice suggest that quality efforts should focus on maintaining the strength of rapid triage and diagnosis (door-to-imaging) while actively addressing delays in drug administration to reduce DNT. At the health policy level, the continued use of quality registries like RES-Q is fundamental for continuous improvement and supports the expansion of IVT protocols and the creation of national stroke networks, ensuring equitable access to this vital therapy.

## Data Availability

Publicly available datasets were analyzed in this study. This data can be found here: https://stroke.qualityregistry.org/.
